# Berberine inhibited metastasis through miR-145/MMP16 axis in vitro

**DOI:** 10.1186/s13048-020-00752-2

**Published:** 2021-01-06

**Authors:** Jie Li, Songlin Zhang, Lei Wu, Meili Pei, Yu Jiang

**Affiliations:** 1 Department of Pathology, the First Affiliated Hospital of Xi’an Jiaotong University, 277 West Yanta Road, 710061 Xi’an, Shaanxi China; 2 Department of Structural Heart Disease, the First Affiliated Hospital of Xi’an Jiaotong University, Xi’an, China; 3 Department of Gynecology and Obstetrics, the First Affiliated Hospital of Xi’an Jiaotong University, Xi’an, China

**Keywords:** Berberine, miR-145, MMP16, Ovarian cancer

## Abstract

Ovarian cancer is the first leading cause of death in gynecological cancers. The continuous survival and metastasis of cancer cells are the main causes of death and poor prognosis in patients with ovarian cancer. Berberine is an effective component extracted from the rhizomes of coptis chinensis and phellodendron chinensis. In our study, we aim to explore the molecular mechanism underlying the regulation of proliferation, migration and invasion by berberine in ovarian cancer cells. CCK8 assay was used for detection of proliferative capacity of SKOV3 and 3AO cells. Wound healing assay was used to estimate cell migration and transwell assay was used to assess cell invasion. The mRNA expression of miR-145 and MMP16 were examined by quantitative real-time polymerase chain reaction (qRT-PCR). The protein level of MMP16 was detected by western blot analysis. In addition, luciferase reporter assays were used to demonstrate MMP16 was a target of miR-145. The results demonstrated berberine inhibited proliferation, migration and invasion, promoted miR-145 expression, and decreased MMP16 expression in SKOV3 and 3AO cells. MMP16 was a target of miR-145. Moreover, downregulation of MMP16 contributed to the inhibition of proliferation, migration and invasion by berberine. Together, our results revealed that berberine inhibited proliferation, migration and invasion through miR-145/MMP16 in SKOV3 and 3AO cells, highlighting the potentiality of berberine to be used as a therapeutic agent for ovarian cancer.

## Introduction

Epithelial ovarian cancer (EOC) is the first leading cause of death in gynecological cancers [1, 2]. The continuous survival and metastasis of cancer cells are the main causes of death and poor prognosis in patients with ovarian cancer. Therefore, inhibiting the proliferation and metastasis of cancer cells is of great significance for the treatment of ovarian cancer.

microRNAs(miRs) are a class of non-coding RNAs that are 20 to 25 nucleotides in length [3]. microRNAs suppress their target genes by binding messenger RNAs at their 3’-untranslated regions (3’-UTRs), either inhibiting protein translation or causing mRNA cleavage. miRs play important regulatory roles in cancer development and progression via manipulating cell growth, proliferation, differentiation and cell death [4–6]. The first discovery of miR-145 was based on its homology with a proven microRNA in mice, which was subsequently demonstrated to be significantly lower in human colon cancer [7, 8]. MiR-145 is located on chromosome 5q32-33 and has a length of 4.08 kb [9]. So far, a large number of oncogenes have been identified as target genes of miR-145. These target genes are involved in regulating many biological functions, including proliferation, migration, differentiation, angiogenesis and so on [10, 11]. Studies show that miR-145 is usually down-regulated in various types of cancer, including ovarian cancer [12, 13]. Published data confirmed that miR-145 may be a tumor suppressor gene expressed in a variety of tumor tissues (including ovarian cancer, cervical cancer, breast cancer and colorectal cancer), and its expression level is significantly lower than normal tissues [14–16]. Our previous studies have shown that miR-145 inhibits ovarian cancer metastasis by blocking epithelial-mesenchymal transition in ovarian cancer cells [11].

Extracellular matrix transfer and basement membrane degradation are important for invasion and metastasis. At present, there is much evidence that MMPs play an important role in tumor invasion and metastasis, and the expression of MMPs is related to the poor prognosis of a series of human cancers [17–20]. However, the mechanism of MMP16 in ovarian cancer remains unclear.

Berberine, the main alkaloid component in Huang Lian, has long been utilized as a traditional Chinese medicine in China. In recent years, many studies have shown that berberine can inhibit many kinds of cancer cells. Yan et. al [21] found that berberine could inhibit proliferation and induce apoptosis of bladder cancer cells. Mahata et. al [22] found that berberine could inhibit HPV by inhibiting catalytic protein-1 and blocking carcinoembryonic associated proteins E6 and E7 induced by HPV, thus inhibiting cervical cancer. In addition, the results suggested that berberine could modulate the sensitivity of cisplatin via regulating miR-21/PDCD4 axis in the ovarian cancer cells [23]. In this study, we have discovered for the first time that berberine could promote the expression of miRNA-145, thus inhibiting the expression of MMP16 and the progression of ovarian cancer, promising a novel natural agent for anti-ovarian cancer therapy.

## Materials and Methods

### Cell culture and berberine treatment

The human ovarian cancer cell line SKOV3 was obtained from the Shanghai Cell Bank of Chinese Academy of Sciences (Shanghai, China), 3AO was from the Shandong Academy of Medical Sciences (Jinan, China). Cells were maintained in RPMI 1640 supplemented with 10% newborn bovine serum (GIBCO, Grand Island, 108 NY, USA). The cells were exposure to 40 µM of berberine (for SKOV3) or 80 µM of berberine(for 3AO).

### Quantitative real-time PCR (qRT-PCR)

Total RNA was extracted from cultured cells using Trizol(Invitrogen, Carlsbad, CA, USA)according to the manufacturer’s instructions. According to the OD260/OD280 ratio, the quality of RNA was estimated. The ratio between 1.8 and 2.0 met the experimental requirements. cDNA synthesis was conducted using RevertAid first strand cDNA synthesis Kit (Thermo Fisher Scientific Inc., Waltham, MA, USA) according to the manufacturer’s instructions. Quantitative real-time PCR was performed using a SYBR Premix Ex Taq™ II kit (Takara, Dalian, China). miR-145 were normalized to U6, while MMP16 were normalized to the gene β-actin. Relative gene expression was calculated automatically using 2^−ΔΔCt^. The following primer sequences of human genes were used:

MMP16 forward: 5′- GGACAGAAATGGCAGCACAAGC-3′;

MMP16 reverse: 5′- CATCAAAGGCACGGCGAATAGC-3′;

β-actin forward: 5’-TCCCTGGAGAAGAGCTACGA-3’;

β-actin reverse: 5’-AGCACTGTGTTGGCGTACAG-3’.

### Western blot

Total proteins were extracted by RIPA lysis buffer(Roche, Indianapolis, IN, USA) and 1 mM PMSF on ice, proteins were separated by SDS-PAGE and then transmembrane. 5% skimmed milk was sealed at room temperature for 2 hours, and then incubated overnight at 4 °C with rabbit anti-human MMP16(1:500, Cell Signaling Technology, Danvers, MA, USA) and mouse anti-human β-actin(1:1000, Cell Signaling Technology, Danvers, MA, USA). TBST membrane was washed for 8 minutes and 5 times, the blots were incubated with horse radish peroxidase (HRP)-conjugated goat anti-rabbit or anti-mouse IgG for 2 hours. The TBST membrane was washed 5 times for 8 minutes each time.

### Plasmid transfection

The human MMP16 expression vector(#22,694) were obtained from Addgene. SKOV3 and 3AO cells were seeded into 6-well plates until 70%-90% confluency and transiently transfected with vector or empty vector 3 µg per well using the X-treme GENE HP DNA Transfection Reagent (Roche, Indianapolis, IN, USA) following the manufacturer’s protocol.

### MicroRNA mimic or inhibitor transfection

miR-145 mimic(miR-145 inhibitor) and negative control were purchased from Ribo-Bio Co. Ltd. (Guangzhou, China). SKOV3 and 3AO cells were transiently transfected with 60 nM miR-145 mimic (SKOV3) and 80 nM miR-145 mimic (3AO) or 100 nM miR-145 inhibitor(SKOV3) and 120 nM miR-145 inhibitor(3AO) or negative control using the X-treme GENE siRNA Transfection Reagent (Roche, Indianapolis, IN, USA) following the manufacturer’s protocol.

### CCK8 assay

Cells in logarithmic growth phase were inoculated into 96-well plates with 5000 holes per hole, 100µL of culture medium was added into each hole and incubated overnight in a 37℃,5% CO_2_ incubator, then add berberine for 48 hours. 10µL CCK8(7Sea, Shanghai, China) was added to each pore and incubated at 37℃ for 4 hours. The absorbance value of each pore OD 450 was determined by enzyme labeling(PerkinElmer, Waltham, MA, USA).

### Wound healing assay

The cells in logarithmic growth phase were taken, when the cell density reached about 90%, covered with the bottom of the 6-well plate, and scratched perpendicular to the horizontal line behind the gun head as far as possible. The gun head should be vertical and not inclined. The cells were washed with PBS three times, the scratched cells were removed, serum-free medium was added, and 0-hour photographs were taken at the same time; the cells were cultured with 37℃ and 5% CO2, photographs were taken again after 24 hours. The measurement of cell scratch was done by Image J.

### In vitro Matrigel invasion assay

A total of 5 × 10^5^ cells in 100 µl serum-free medium were added into millicells (Millipore Co., Bedford, MA, USA) with Matrigel (Becton Dickinson Labware, Bedford, MA, USA) coated. The cells were cultured in a 5% CO2 incubator at 37℃ for 24 hours, transwell was removed, the cells were carefully cleaned with PBS, fixed with 70% ice ethanol solution for 1 hour, and stained with 0.5% crystal violet dye. Place it at room temperature for 20 minutes, wash it with PBS, wipe the unmigrated cells on the upper side of the room with clean cotton ball, and take photos under the microscope.

### Luciferase reporter assay

Cells were co-transfected with pRL-TK vector (20 ng), wild-type or mutant reporter vectors (180 ng), along with miR-145 mimic or negative control at a final concentration of 20 nM using the X-treme GENE siRNA Transfection Reagent. 24 h after transfection, the relative firefly luciferase activity (normalized to Renilla luciferase activity) was measured using a dual-luciferase reporter gene assay system (Promega, Madison, WI, USA), and results were depicted as the percentage change over the respective control.

### Statistical analysis

All experiments were performed at least in triplicate, and each experiment was independently performed at least 3 times. The graphical presentations were performed using GraphPad Prism 5.0. Data were presented as the means ± SE and were analyzed using SPSS 22.0 software (Chicago, IL, USA). Statistical differences were tested by Chi-square test, two-tailed t-test, or Fisher’s Exact test. Differences were considered significant at *P* < 0.05 (*) or highly significant at *P* < 0.001 (**).

## Results

### Berberine promotes miR-145 expression in ovarian cancer cells

**Fig. 1:**
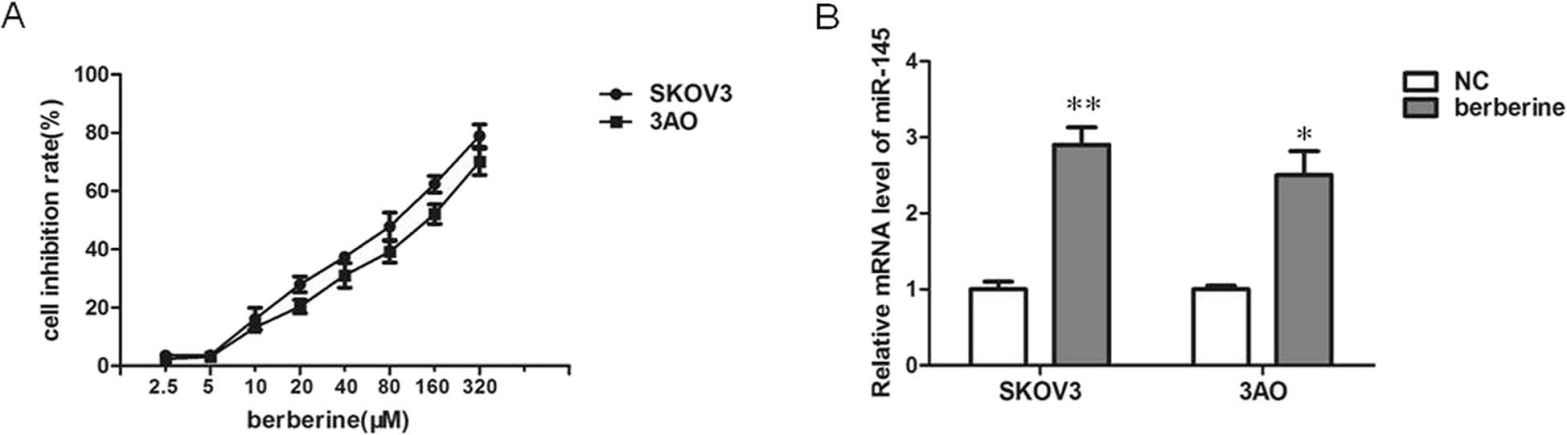
Berberine promotes miR-145 expression in ovarian cancer cells. **a **CCK8 assay results showed that berberine could inhibit the growth of SKOV3 and 3AO cells in a dose-dependent manner. **b** qRT-PCR results showed berberine increased miR-145 expression. *P* < 0.05, **P < 0.01, *t*-test

We first examined the effect of berberine on cell viability of the two human ovarian cancer cell lines SKOV3 and 3AO through CCK8 assays. Berberine inhibited cell growth in a dose-dependent manner, giving rise to IC50 (inhibitory concentration at which 50% cell viability is inhibited) values of 78.52 µM and125.8 µM, respectively, for SKOV3 and 3AO cells 48 h after treatment (Fig. 1a). We then examined the effect of berberine on the expression of miR-145. The results showed that berberine could promote the expression of miR-145 (Fig. 1b).

### Berberine inhibits proliferation, migration and invasion through miR-145 in SKOV3 and 3AO cells

**Fig. 2:**
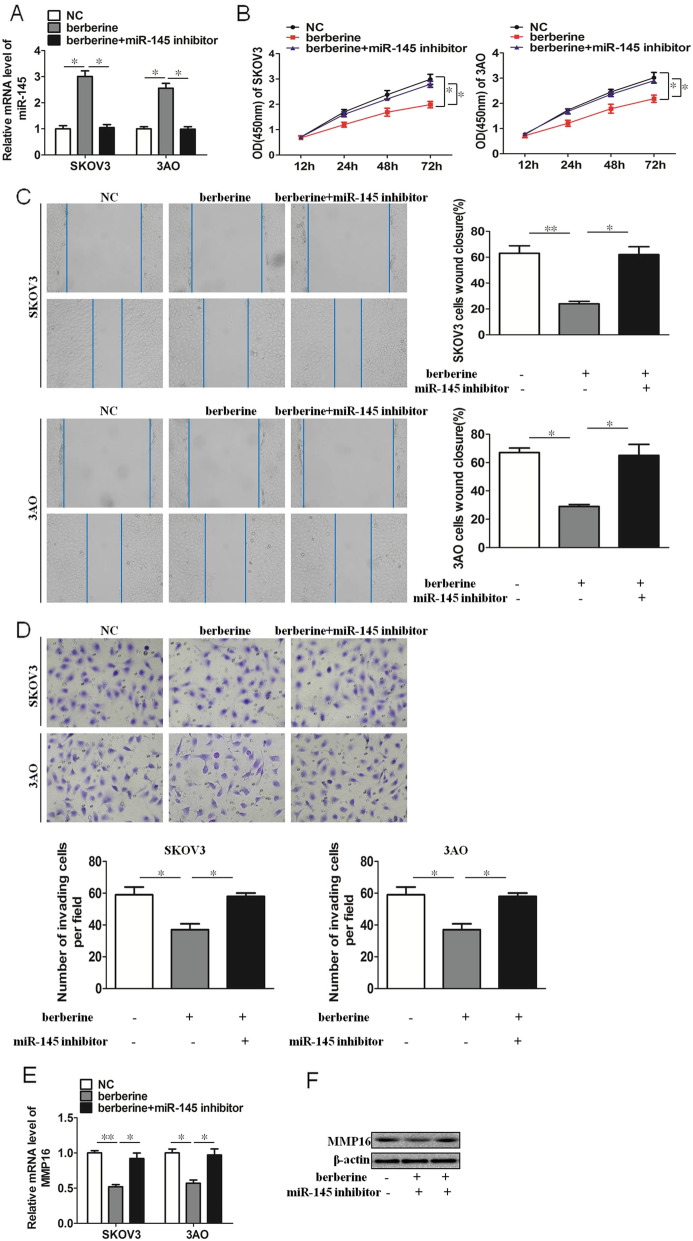
Berberine inhibits proliferation, migration and invasion through miR-145 in SKOV3 and 3AO cells. **a** qRT-PCR results showed berberine promoted miR-145 expression, and this promotion effect was offset by transfection of miR-145 inhibitor. **b** CCK8 assay results showed berberine inhibited proliferation of SKOV3 and 3AO cells, and this inhibition was blocked by downexpression of miR-145. **c **Wound healing assay showed berberine inhibited migration of SKOV3 and 3AO cells, and this inhibition was blocked by downexpression of miR-145. **d** Transwell assay showed berberine inhibited invasion of SKOV3 and 3AO cells, and this inhibition was blocked by downexpression of miR-145(200×). **e** qRT-PCR results showed berberine inhibited the expression of MMP16, and the inhibition of MMP16 was counteracted by downexpression of miR-145. **f **Western blot assay showed berberine inhibited the expression of MMP16, and the inhibition of MMP16 was counteracted by downexpression of miR-145. *P* < 0.05, **P < 0.01, *t*-test

Berberine promoted the expression of miR-145, which can be reversed by miR-145 inhibitor transfection (Fig. 2a). We then examined the proliferation, migration and invasion of ovarian cancer cells. The results showed that berberine could inhibit the proliferation, migration and invasion of SKOV3 and 3AO cells, but this inhibition was blocked by downregulation of miR-145 (Fig. 2b-d). Futhermore, we examined the expression of MMP16, the results showed that berberine could inhibit the expression of MMP16, and the inhibition of MMP16 was counteracted after the transfection of miR-145 inhibitor (Fig. 2e,f). These results demonstrated that upregulation of miR-145 contributed to the inhibition of proliferation, migration and invasion by berberine in SKOV3 and 3AO cells.

### MMP16 is a target of miR-145

**Fig. 3:**
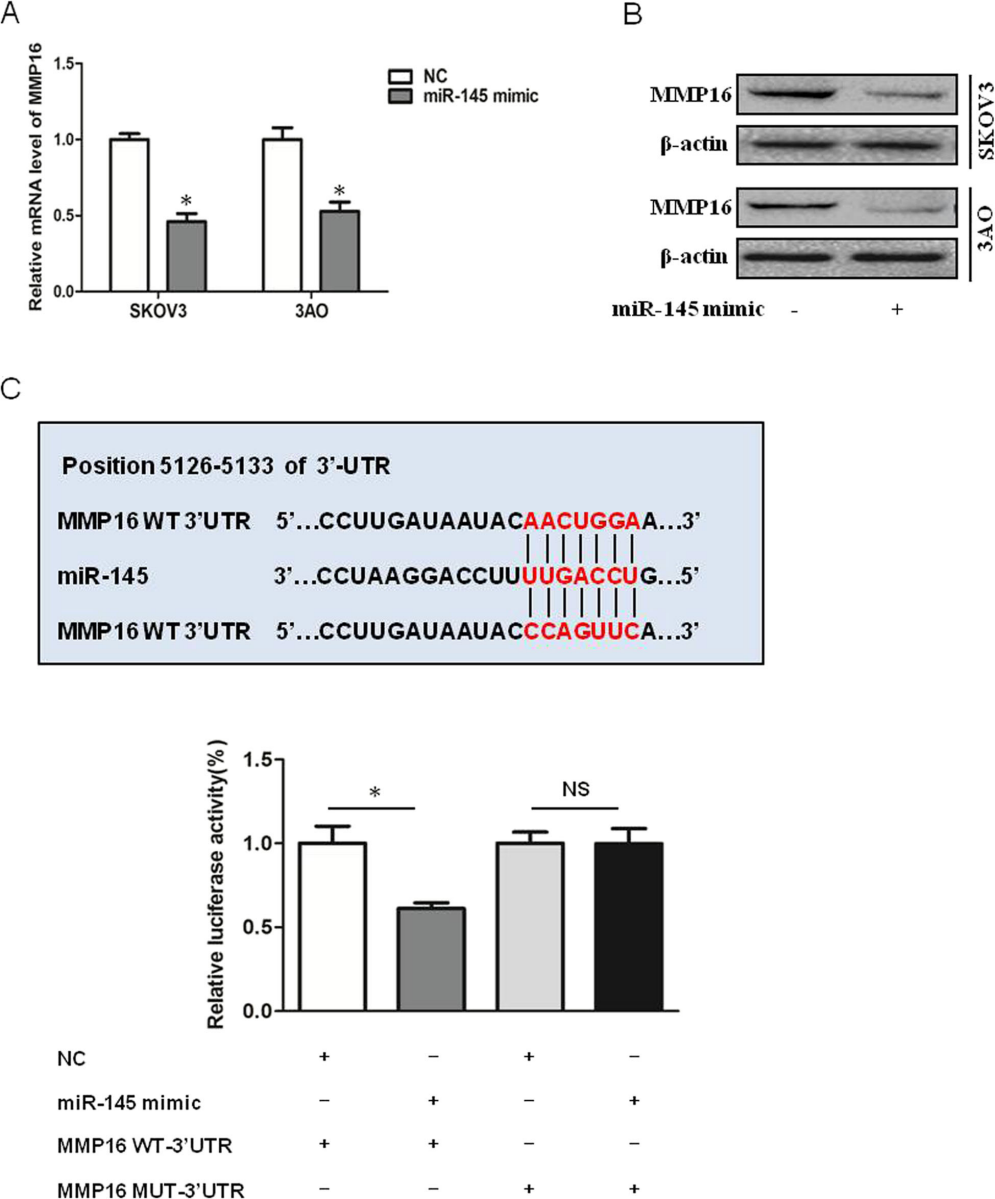
MMP16 is a target of miR-145. **a** qRT-PCR results showed overexpression of miR-145 inhibited MMP16 in SKOV3 and 3AO cells. **b** Western blot results showed overexpression of miR-145 inhibited MMP16 in SKOV3 and 3AO cells. **c **The luciferase reporter assay showed miR-145 directly. *P* < 0.05, **P < 0.01, *t*-test

Next we explored the target of miR-145 to inhibit ovarian cancer progression. We first predicted putative target genes of miR-145 by searching the TargetScan database (release 5.1, http://www.targetscan.org/), and MMP16 was chosen to be experimentally verified. qRT-PCR results demonstrated overexpression of miR-145 inhibited MMP16 expression on both mRNA (Fig. 3a) and protein levels (Fig. 3b). In addition, The luciferase reporter assay showed that luciferase activity was significantly inhibited in cells co-transfected with miR-145 mimic and MMP16 WT-3’UTR vector, while no changes of luciferase activity were detected in cells transfected with miR-145 mimic and luciferase reporter plasmids containing the mutant seeding sequence (Fig. 3c). These results clarified miR-145 targeted MMP16 directly.

### miR-145 inhibits proliferation, migration and invasion of SKOV3 and 3AO cells by targeting MMP16

**Fig. 4:**
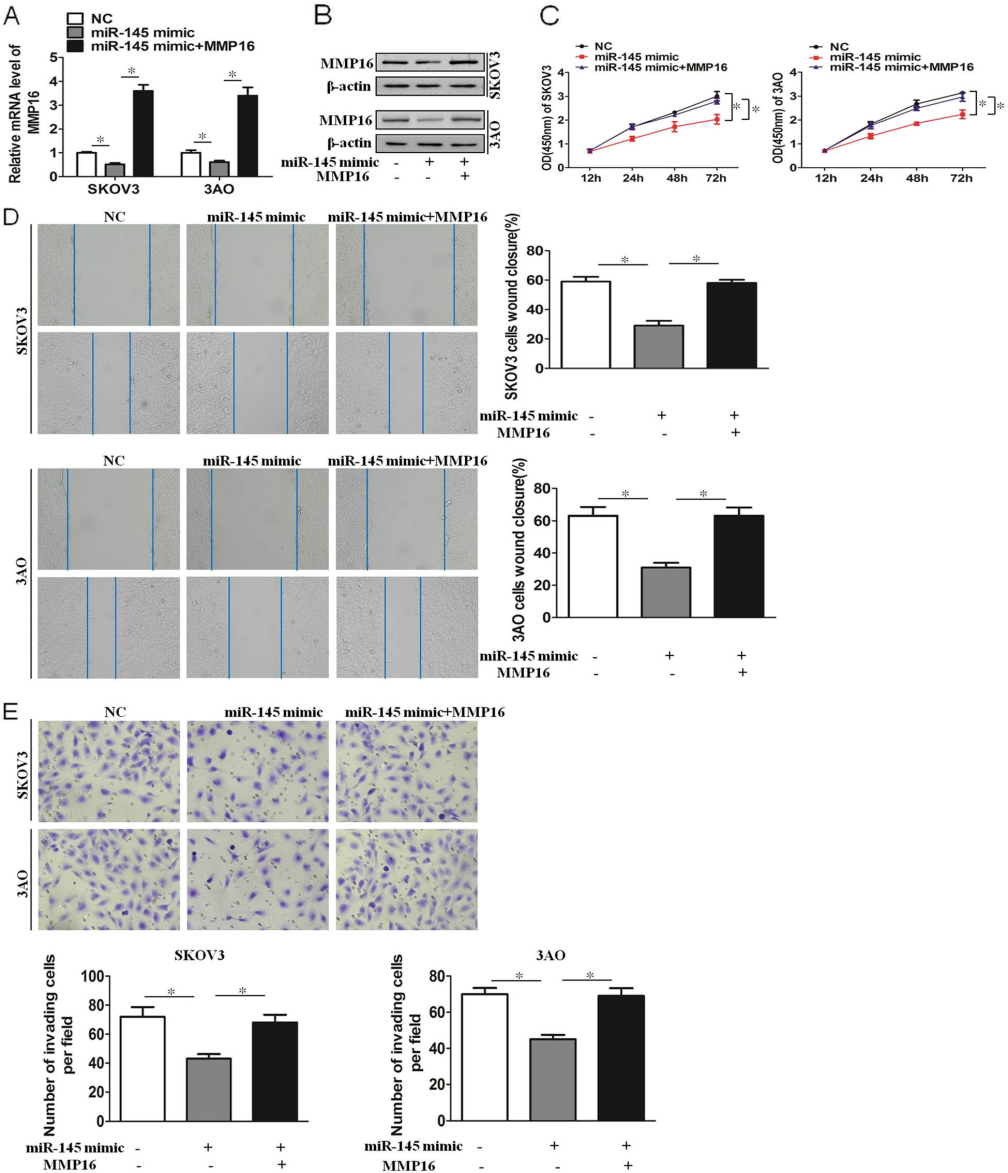
miR-145 inhibits proliferation, migration and invasion of SKOV3 and 3AO cells by targeting MMP16. **a** qRT-PCR results showed overexpression of miR-145 inhibited MMP16 expression, and MMP16 expression was increased by transfection MMP16 plasmid. **b **Western blot results showed overexpression of miR-145 inhibited MMP16 expression, and MMP16 expression was increased by transfection MMP16 plasmid. **c** CCK8 assay results showed miR-145 inhibited proliferation, and the inhibition effect was reversed by MMP16 overexpression. **d **Wound healing assay showed miR-145 inhibited migration, and the inhibition effect was reversed by MMP16 overexpression. **e **Transwell assay showed miR-145 inhibited invasion, and the inhibition effect was reversed by MMP16 overexpression(200×). *P* < 0.05, **P < 0.01, *t*-test

In SKOV3 and 3AO cells, the expression of MMP16 was decreased by transfection of miR-145 mimic, and the downxpression of MMP16 was reversed after transfection of MMP16 plasmid (Fig. 4a,b). Next, we explored the proliferation, migration and invasion of ovarian cancer cells induced by miR-145. The results showed miR-145 inhibited proliferation, migration and invasion of SKOV3 and 3AO cells, and the inhibition effects were reversed by overexpression of MMP16 (Fig. 4c-e). These results demonstrated that miR-145 inhibited proliferation, migration and invasion of SKOV3 and 3AO cells by targeting MMP16.

## Discussion

**Fig. 5:**
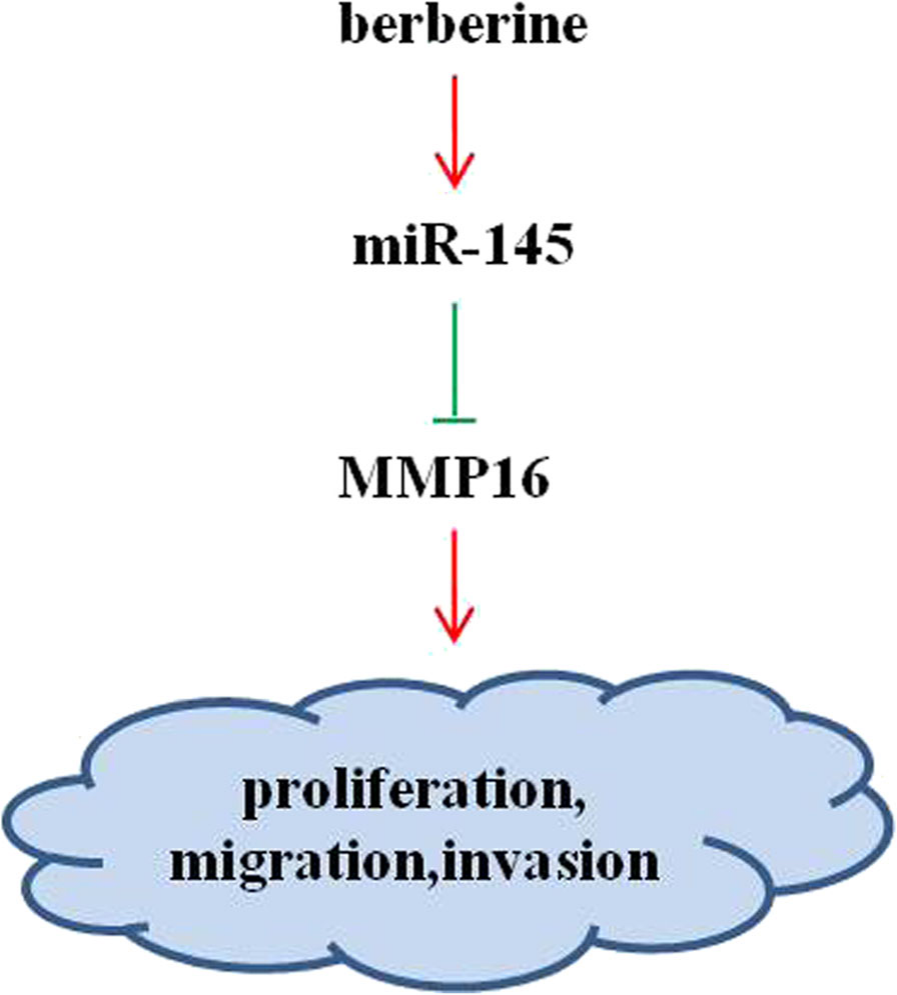
Schematic representation of the mechanism for berberine‑regulated ovarian cancer progression

Ovarian cancer is a lethal disease of increasing incidence worldwide, and it is the first leading cause of death from gynecological cancers [24]. The persistent survival and metastasis of cancer cells are the main causes of death and poor prognosis in patients with ovarian cancer. In this regard, it represents an attractive therapeutic approach anticancer drug discovery to search pharmacologically active ingredients from natural sources such as Chinese herbs to inhibit tumor growth and metastasis. In present study, we have demonstrated for the first time that berberine inhibits proliferation, migration and invasion through miR-145/MMP16 in SKOV3 and 3AO cells (Fig. 5).

Berberine is an effective component extracted from the rhizomes of Coptis chinensis and Phellodendron chinensis [25]. Berberine has a long history in traditional Chinese medicine, mainly used for heat clearing, detoxification, anti-infection and so on [26, 27]. With the in-depth study of other functions of berberine, it has been found that berberine also has pharmacological effects such as lowering blood lipid, lowering blood sugar, anti-arrhythmia, expanding coronary vessels and anti-hypertension [25]. In recent years, a large number of studies have shown that berberine has anti-tumor effect, and can participate in the anti-tumor process of liver cancer [28], lung cancer [29], colon cancer [30], bladder cancer [31], and other malignant tumors. The anti-tumor mechanism of berberine is still under constant study. Its main mechanism is to induce cells apoptosis, stagnation of tumor cell cycle, inhibition of tumor cell metastasis, inhibition of tumor angiogenesis. In present study, our results revealed that berberine inhibited proliferation, migration and invasion in SKOV3 and 3AO cells. This is consistent with previous studies in other tumors.

Adhesion, invasion and metastasis are important biological characteristics of tumors [32]. They are the direct causes of loss of operation opportunities or progression of tumors. Berberine can inhibit the invasion and metastasis of tumors in many ways [33, 34]. Matrix metalloproteinases (MMPs) are closely related to the adhesion, invasion and metastasis of tumors [35]. MMPs can cause the degradation of extracellular matrix of tumors and provide conditions for the invasion and metastasis of tumors [36, 37]. This is also the initial step of metastasis and invasion of tumors. In our study, we found berberine inhibited the expression of MMP16 and thus inhibited the proliferation, migration and metastasis of ovarian cancer cells. Previous studies have shown that berberine could significantly inhibit the level of urokinase type plasminogen activator and MMP-9 in hepatoma cells, inhibit the degradation of tumor extracellular matrix, and thus inhibit tumor cells invasion and metastasis [38]. However, the relationship between berberine and MMP16 has not been reported. Samely, previous studies on berberine and ovarian cancer mostly focused on the relationship between berberine and drug resistance of ovarian cancer [39, 40], but few reports on metastasis of ovarian cancer. Our study improved the molecular mechanism of berberine inhibiting the progression of ovarian cancer.

Our previous studies have shown that miR-145 can inhibit the progression of ovarian cancer by inhibiting the Warburg effect [41], glutamine metabolism [42] and epithelial-mesenchymal transition [11]. In present study, we found berberine could increase miR-145 expression, and miR-145 targeted MMP16 directly, these results suggest that miR-145 may be a target of berberine in the treatment of ovarian cancer.

## Conclusions

In conclusion, our data showed that berberine promoted miR-145 expression and decreased MMP16 expression, thus inhibiting proliferation, migration and metastasis of ovarian cancer SKOV3 and 3AO cells. Our work reported here lays ground for necessary additional studies of its clinical use as a new anti-cancer drug for the treatment of various metastatic diseases including ovarian cancer.

## Data Availability

The datasets during and/or analysed during the current study available from the corresponding author on reasonable request.
